# Flavonoid contributors to bitterness in juice from *Citrus* and *Citrus* hybrids with/without *Poncirus trifoliata* in their pedigree^☆^

**DOI:** 10.1016/j.fochx.2025.102289

**Published:** 2025-02-18

**Authors:** Kristen A. Jeffries, Zhen Fan, Matthew Mattia, Ed Stover, Elizabeth Baldwin, John A. Manthey, Andrew Breksa, Jinhe Bai, Anne Plotto

**Affiliations:** a USDA-ARS, U.S. Horticultural Research Laboratory, 2001 South Rock Road, Fort Pierce, FL 34945-3030, USA; b Horticultural Sciences Department, IFAS Gulf Coast Research and Education Center, University of Florida, Wimauma, FL 33598, USA; c USDA-ARS, 800 Buchanan Street, Albany, CA 94710, USA

**Keywords:** Bitter, GC–MS, LC-MS/MS, Flavonoids, Limonoids, *Citrus*, *Poncirus trifoliata*

## Abstract

Bitterness and off-flavors are problematic in some citrus genotypes, particularly *Citrus* hybrids with *Poncirus trifoliata* in their pedigrees that have shown tolerance to the devastating citrus greening disease. Comprehensive chemical profiling combined with sensory analysis of bitterness were used to determine bitter compounds in citrus juice. A selection of genotypes including orange, grapefruit, pummelo, tangelo, mandarin hybrids with and without *P. trifoliata* in their pedigrees, as well as pure *P. trifoliata,* were analyzed due to their broad range in bitterness intensity. Widely targeted LC-MS/MS analysis of flavonoids and limonoids confirmed the role of limonin, nomilin, neohesperidin and poncirin in bitterness perception. Other flavonoids, mainly rhoifolin, apigenin, and tricin also correlated with bitterness. Notably, rhoifolin was more strongly correlated with bitterness than the previously known bitter compounds. Identifying the compounds that contribute to bitterness in citrus is a crucial first step for future breeding efforts aimed at reducing these compounds biosynthetically.

## Highlights

• Bitterness of eleven citrus accessions ranged from 1.5 to 11.2 on a 0–15 scale.
• GC–MS and LC-MS/MS were used to profile volatile and non-volatile compounds.
• Some volatile monoterpenes and sesquiterpenes were correlated with bitterness.
• Rhoifolin, apigenin and tricin flavonoids were correlated with bitterness in citrus.
• Identified bitter compounds can be used as negative biomarkers in citrus breeding.

## 1 Introduction

Bitterness in citrus juice is undesirable and researchers have been studying the chemical components responsible for bitterness in citrus for many years (Emerson, 1948; Horowitz & Gentili, 1963). More recently, bitterness in citrus has become a significant quality issue as Huanglongbing (HLB), also known as citrus greening disease, has devastated Florida's citrus agriculture. HLB is the primary cause for the 90 % drop in production of Florida citrus since its discovery in 2005 (USDA-NASS, 2024) and also causes bitterness and off-flavors in juice made with fruit harvested from HLB-affected trees (Baldwin et al., 2010). Another source of bitterness in citrus juice is inherent delayed bitterness, a quality issue for some cultivars that pre-dates the HLB era. While moderate amounts of bitterness can be perceived as a positive attribute in fruit such as grapefruit, excessive bitterness is unpleasant and undesirable (L. Li et al., 2023). There have been efforts to develop postprocessing methods to debitter citrus juices, however this is complicated and a substantial expense for processors as bitterness is not caused by a single chemical compound, but rather many compounds acting synergistically or antagonistically (Purewal & Sandhu, 2021).

In citrus, there are well-known compounds that contribute to bitterness, namely two highly oxygenated triterpenes, limonin and nomilin, as well as a flavonoid, naringin, which is produced in pummelo and grapefruit (Purewal et al., 2021). There are large numbers of limonoids and limonoid glycosides endogenous to citrus and although there has been work to elucidate the enzyme responsible for glycosylation of limonoids (Cui et al., 2020; Kita et al., 2000), their biosynthetic genes are still largely unknown. As fruit maturation proceeds, a natural debittering occurs as bitter limonoid aglycones are glycosylated. Upon juicing, delayed bitterness occurs when limonoate A-ring lactone (LARL), which is highly abundant in segment membranes, is converted to bitter limonin as it is released into an acidic environment (Maier & Beverly, 1968).

Citrus juices are very rich in polyphenolic compounds and some of these non-volatile compounds, such as flavonoids, can contribute to the complex flavor. The flavonoid backbone can be highly substituted, yielding a wide variety of compounds with diverse biological activities (L. Li et al., 2023). Some flavonoids are tasteless while others are intensely bitter or even blockers of bitterness (Z. Wang, Gmitter, Grosser, & Wang, 2022). Additionally, interconversion between flavonoids occurs, such as naringin (naringenin-7-O-neohesperidoside) being hydrolyzed via naringinase into less bitter prunin (naringenin-7-glucoside), which may be further hydrolyzed into the tasteless aglycone, naringenin (Ribeiro & Ribeiro, 2008). Understanding the enzymatic basis of bitterness in citrus was proposed to be due to conjugation to specific moieties, such as glycosylation with neohesperidose or rutinose by 1,2-rhamnosytransferase or 1,6-rhamnosytransferase, respectively (Frydman et al., 2013). However, there is little consistency regarding chemical structure related to bitterness. This is likely due to the promiscuity of the human bitter taste receptors. Of the twenty-five bitter taste receptors, a number of flavonoids have been predicted by modeling to activate TAS2R14 and TAS2R39 (Roland et al., 2013). From this work, it was suggested that hydroxyl-rich flavonoids are more likely to activate these two receptors. Additionally, polymethoxylated flavones, tangeretin and nobiletin, can be perceived by TAS2R14 and TAS2R46 (Kuroda et al., 2016).

It is well known that sugars and other non-volatile compounds can enhance or mask bitterness (Walters & Roy, 1996), but the interaction between taste and retronasal olfactory perception of volatile compounds is less understood. Researchers have shown that various sets of volatile compounds, according to the type of fruit, can suppress bitterness in foods, independent of sugars (Schwieterman et al., 2014; Spence, 2022). In oranges, 14 volatile compounds significantly associated with bitterness suppression were found to suppress bitterness by increasing sweetness (Bartoshuk et al., 2018). While it has been shown that volatile compounds can enhance sweetness (Tieman et al., 2012), future research is needed to understand the complex interaction of odor-induced taste enhancement or suppression.

Polymethoxylated flavones, tangeretin and nobiletin, have been reported to be important contributors to bitterness in citrus peel, but in juice these compounds are not abundant enough to significantly impart bitterness (Kiefl et al., 2018). In 2018, a study of off-flavors due to HLB-affected orange juice concluded that bitterness was imparted by unidentified non-volatile compounds other than limonin, nomilin, tangeretin, or nobiletin (Dala Paula et al., 2018). It was suggested that the unidentified compounds could be derivatives of hydroxycinnamates. In a more recent study, the bitterness in hybrids of *Citrus* x *Poncirus trifoliata* was suggested to be due to unidentified flavonoids, instead of limonin or nomilin since their concentrations were not above their respective taste recognition thresholds (Deterre et al., 2021). Interest in *Citrus* hybrids with *P. trifoliata* introgression has increased due to their tolerance to HLB. Recently, some of their off-flavors have been reported to be due to the flavonoid, linarin (Jeffries et al., 2024)

In order to identify compounds that are correlated with bitterness in citrus, a broad selection of genotypes were selected for this study, including oranges, grapefruit, pummelo, tangelo, mandarin hybrids without *P. trifoliata* in their pedigrees (referred to as mandarin hybrids herein), pure *P. trifoliata*, and complex *Citrus* hybrids with *P. trifoliata* introgression into a largely mandarin-derived pedigree (referred to as *Poncirus* hybrids herein). While there have been some studies for citrus that correlated sensory panels with limonoids and flavonoids, the number of secondary metabolites quantified were limited to the known bitter limonoids (limonin and nomilin) and some flavonoids (tangeretin, nobiletin, and sinensetin) (Plotto et al., 2017; Raithore et al., 2016). To comprehensively analyze the complex juice samples multiple analytical methods are utilized herein, including titration to measure acids, refractometry to measure sugars, GC–MS to profile volatile compounds, and LC-MS/MS to widely target flavonoids and limonoids. Understanding the compounds contributing to bitterness in citrus could benefit future breeding efforts or debittering method development as selective removal of compounds would be more beneficial than removing all compounds that give citrus juices their health benefits.

## 2 Materials and methods

### 2.1 Fruit material and juicing

Fruits were harvested during the 2021–2022 season from mature trees (genotypes and classifications included in Table 1 and Table 2) grown at the USDA, ARS Research Farm in Fort Pierce, FL or the USDA, ARS, Whitmore Citrus Research Foundation Farm in Groveland, Lake County, FL. At maturity, fruits were harvested to include a wide range of bitter samples. Following washing and sanitizing (Deterre et al., 2021), fruits were batched into four biological replicates with equal numbers of fruit (five to eight, depending on size). The fruit were manually juiced with a reamer-type juicer (Vinci™ Hand Free Juicer, Vinci® Housewares, La Mirada, CA, USA) and juice samples were stored at −20 °C. Juice samples analyzed by LC-MS/MS were stored at −80 °C.

**Table 1:** Bitterness ratings and panelists' descriptive comments of selected Citrus/*Poncirus* hybrids.

Genotype	Citrus type	Harvest Date	Bitter Rating^z^	Panelists' Comments
FF-1-5-35	Mandarin hybrid	12/2021	10.2a	Very bitter, “poncirus,” lingering, metallic, low sweetness, lacking orange flavor
‘Wekiwa’	Tangelo	01/2022	5.2de	Balanced sweetness and bitterness, pumpkin, floral, grapefruit, “poncirus”
‘Melogold’	Pummelo	03/2022	11.2a	Very bitter, grapefruit, lingering, pummelo
FF-1-5-35	Mandarin hybrid	01/2022	8.4b	Bitter, pumpkin, earthy, bland, astringent, lacking orange flavor
FF-1-85-124	Poncirus hybrid	01/2022	10.2a	Bitter, sour, “poncirus,” gasoline, cardboard, citrus leaf, spicy, lemon, low sweetness
FF-5-6-36	Mandarin hybrid	11/2021	6.5 cd	Balanced sweetness and bitterness, grapefruit, “poncirus,” floral, peel oil
‘US119’	Poncirus hybrid	11/2021	4.6e	High off-flavor, “poncirus,” some bitterness, green, pine, astringent, acrid
‘Glen Navel’	Orange	12/2021	7.6bc	Very bitter, low sweetness, green, peel oil, sour
FF-1-84-2	Mandarin hybrid	01/2022	1.9f	Sweet, low bitterness, mandarin, pleasant, sour
FTP-6-32-67	Mandarin hybrid	01/2022	2.8f	Sweet, slight bitterness, sweet pea, pumpkin, floral, fruity-non citrus
‘Valencia’	Orange	04/2022	1.5f	Very sour, slightly sweet, orange, off-flavor, fruity

z Bitter intensity ratings were on a linear scale with marks at every unit, 0 to 15. Means followed with the same letter are not significantly different by the Fisher's Least Significant Difference test (LSD) test for multiple comparisons (*P* < 0.05).

**Table 2:** Soluble solids content (SSC), titratable acidity (TA) and SSC/TA of selected Citrus/*Poncirus* hybrids^z^.

Genotype	Classification	Harvest Date	SSC (%)	TA (%)	SSC/TA
FF-1-5-35	Mandarin hybrid	12/2021	10.51 cd	0.61 h	17.38b
FF-1-5-35	Mandarin hybrid	01/2022	11.92b	0.58 h	20.52b
‘US119’	*Poncirus* hybrid	11/2021	10.89bc	0.67gh	16.27b
‘Glen Navel’	Orange	12/2021	10.66bc	0.62 h	17.28b
FTP-6-32-67	Mandarin hybrid	01/2022	14.90a	0.51 h	29.46b
‘Wekiwa’	Tangelo	01/2022	11.94b	0.50 h	24.08b
‘Melogold’	Pummelo	03/2022	9.27de	0.65 h	14.29b
FF-1-84-2	Mandarin hybrid	01/2022	14.48a	0.85 fg	17.06b
FF-1-85-124	*Poncirus* hybrid	01/2022	11.63bc	1.19 cd	9.75b
FF-5-6-36	Mandarin hybrid	11/2021	14.06a	0.90ef	15.63b
‘Valencia’	Orange	04/2022	10.71bc	1.03de	10.44b
*P. trifoliata* (small flower)	*P. trifoliata*	11/2021	14.34a	5.98a	2.40b
*P. trifoliata* (large flower)	*P. trifoliata*	11/2021	10.66bc	4.79b	2.23b
Cape Croen Skill	Mandarin hybrid	01/2022	8.69e	0.03i	285.15a
Florida Red	Grapefruit	01/2022	8.56e	1.26c	6.81b

z Numbers followed with the same letter within a column are not statistically different by the Tukey (HSD) test (*P* < 0.05).

### 2.2 Bitter sensory analysis

Eleven trained panelists determined the bitterness rating of each genotype using a linear intensity scale with anchor points at each unit from 0 to 15. During a single session, juice samples were served at 14 °C as 35 mL samples in coded 4 oz. plastic soufflé cups covered with clear plastic lids (Solo® Cups Co., Urbana, IL, USA). A bitter reference standard, 11.5 mg/L quinine hydrochloride (Sigma-Aldrich) in water, was served with the samples and given an intensity value of 7 on the 0 to 15 scale. To minimize saturation from bitterness, samples were prescreened and presented in order from least to most bitter. The sample presented first was ‘Valencia’, followed by FTP-6-32-67, FF-1-84-2, ‘Glen Navel’, ‘US119′, FF-5-6-36, FF-1-85-124, FF-1-5-35 (harvested 01–2022), ‘Melogold’, ‘Wekiwa’, and FF-1-5-35 (harvested 12–2021). In between samples, panelists were provided with salted crackers, water, and a salt rinse (0.5 M NaCl) (Raithore et al., 2020). Panelists recorded their ratings with Compusense® software (Compusense Inc., Guelph, ON, Canada) and had an option to record general comments for each juice sample. This study was conducted within the guidelines of the human subject exemption as stated in 45 CFR 46.104 (d)(6).

### 2.3 Volatile chemical analysis

Volatile profiling was performed via headspace-SPME-GC/MS analysis as described previously (Bai et al., 2016; Fan et al., 2024; Jeffries et al., 2024). In brief, samples (six milliliters of whole juice in a crimp-capped 20 mL vial) were incubated at 40 °C for 30 min, exposed to a SPME fiber (2 cm tri-phase SPME fiber (50/30 μm DVB/Carboxen/ PDMS, Supelco, Bellefonte, PA, USA) for 30 min, and then analyzed with an Agilent 7890 GC equipped with a DB-5 column (60 m × 0.25 mm i.d., 1.00 μm film thickness, J&W Scientific, Folsom, CA, USA) and coupled with a 5975 MS (Agilent Technologies, Palo Alto, CA, USA). A Kovats mixture of C-5 to C-18 n-alkanes was run to determine retention indices (RIs) and to validate the inter-day reproducibility. Volatile compounds were identified using NIST 14 (http://chemdata.nist.gov) and Adams (Adams, 2017) spectral libraries and their identities were confirmed using authentic standards on a DB-Wax capillary column (60 m × 0.25 mm i.d., 0.5 μm film thickness; J&W Scientific). MassHunter Quantitative Analysis software (Agilent, Santa Clara, CA, USA) was used for data analysis and average peak areas of four biological replicates are reported.

### 2.4 Nonvolatile chemical analysis

Flavonoid and limonoid profiling was performed via LC-MS/MS analysis as described previously (Jeffries et al., 2024). In brief, whole juice was extracted with 80 % methanol, filtered with a 0.2 μm nylon syringe, and then injected on a 1290 Infinity II UPLC coupled with a 6470 triple quadrupole MS (Agilent, Santa Clara, CA, USA). Samples were normalized by the addition of an internal standard, mangiferin, at a concentration of 10 μg/mL. Chromatographic separation of analytes was achieved with a ZORBAX RRHD Eclipse Plus C18 column (2.1 × 50 mm, 1.8 μm, Agilent). In dMRM mode, analytes were identified with characteristic MRM transitions and retention times listed in Table S1 in comparison with analytical standards, public databases, and references (Feng et al., 2018; F. Wang et al., 2021; S. Wang et al., 2017). Compound-specific precursor and product ions observed for each collision energy voltage are listed in Table S1. Fragmentor and collision energy voltages as well as the detection polarity were optimized for each compound (Table S1). MassHunter Quantitative Analysis software (Agilent, Santa Clara, CA, USA) was used for data analysis and average relative peak areas of three biological replicates are reported.

The soluble solids content (SSC) was measured with juice supernatant via a refractometer (Atago RX-5000α, Tokyo, Japan). Juice supernatant was collected after centrifuging at 10,000 x *g* for 15 min. The SSC averages of each sample are reported from four biological replicates. The titratable acidity (TA) was measured via a titrator (Dosino model 800, Metrohm, Herisau, Switzerland) by titrating ∼5 g of juice supernatant to pH 8.1 with 0.1 N NaOH. The TA averages of each sample are reported from four biological replicates.

### 2.5 Statistical analyses

Sensory data were analyzed by analyses of variance (ANOVA) using SenPAQ version 6.3 (Qi Statistics, West Malling, UK). A mixed model was used, with “panelist” as a random variable and where the main effects (panelist or sample) were tested against their interaction, with the two replications included in the error term (Lawless & Heymann, 1999).

SSC and TA were analyzed by ANOVA using XLSTAT 2019.1.1 (Lumivero, Denver, CO) and separations of means were performed using the Tukey's honest significance difference (HSD) test at *P* < 0.05.

JMP software Version 16 (JMP Statistical Discovery, Cary, NC, USA) was used to perform 2-way hierarchical clustering analysis, with genotype on the y-axis and non-volatile or volatile compounds on the x-axis. JMP software was used to perform Principal Components Analysis (PCA), to create biplots, and to obtain correlation values. JMP software was used to perform partial least squares (PLS) regression analysis with the bitter rating as the y variable and selected compounds as the x variables. A SIMPLS fit with 2 factors was used to create a correlation loading plot.

## 3 Results and discussion

### 3.1 Sensory evaluation of bitterness

A trained panel rated the intensity of bitterness in eleven Citrus genotypes, including mandarin hybrids with/without *Poncirus* introgression, orange varieties, a pummelo, and a tangelo. Along with their bitterness intensity ratings, panelists were encouraged to record general comments about each genotype. Table 1 shows the samples in the order in which they were presented to panelists, least bitter first and increasing bitterness (bottom to top in Table 1). It appeared that ‘Wekiwa’ was not as bitter as previously judged in a preliminary screening test, while ‘Glen Navel’ was more bitter. ‘Glen Navel’ is known to be highly susceptible to delayed bitterness after juicing and in storage (Jeffries et al., 2024; Maier et al., 1968; Raithore et al., 2016), and it is highly possible that limonin content increased during the 48 h time period from when the juice was prescreened to when it was tasted by the panel. Bitterness intensities ranged from 1.5 in one of Florida's commercial orange cultivars, ‘Valencia’, to 11.2 in the pummelo hybrid, ‘Melogold’. During pre-screening of the juices, it was determined that ‘Cape Croen Skill’, a unique mandarin accession that was collected in Belgian Congo, was too bitter to serve to panelists. The juices of *P. trifoliata* (small and large flower) and ‘Florida Red’ were not served to panelists due to a lack of sample volume. However, these genotypes, as well as ‘Cape Croen Skill’ were analyzed chemically and included in this study.

‘Valencia’, FF-1-84-2 and FTP-6-32-67 had the lowest ratings for bitterness, consistent with a previous study in which the full sensory profile was reported (Jeffries et al., 2024). Panelists commented that these juices were sweet, but also sour (‘Valencia’) with low bitterness. Genotypes with moderate bitterness included ‘US119’, ‘Wekiwa’, FF-5-6-36 and ‘Glen Navel’. Panelists commented that ‘US119’ was astringent and had “poncirus” and “green” or unripe flavor, as previously reported for this genotype (Deterre et al., 2023). The bitterness in ‘Wekiwa’ and FF-5-6-36 was balanced with some sweetness or floral aromatics, while bitterness in ‘Glen Navel’ was enhanced by low sweetness, sourness and peel oil. Genotypes with high bitterness included FF-1-5-35, ‘Melogold’ and FF-1-85-124. FF-1-5-35 was extremely bitter when harvested in December and was slightly less bitter when harvested a month later in January (Table 1). For both harvests of FF-1-5-35, panelists commented that this juice was bitter, lacked orange flavor, and had negative off-flavors. The most bitter *Poncirus* hybrid was FF-1-85-124, which panelists described as bitter, sour, with “poncirus,” gasoline, and cardboard off-flavors. While having the highest bitter intensity rating, panelists did not report negative attributes for ‘Melogold’; rather, they commented that it was bitter with grapefruit and pummelo flavors.

### 3.2 Volatile profiling of genotypes

**Fig. 1:**
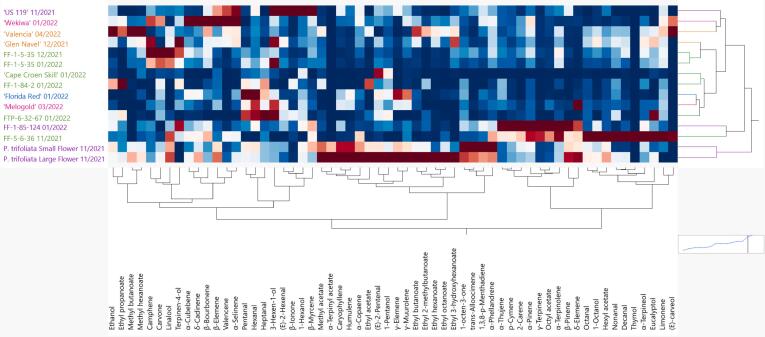
Hierarchical clustering (2-way) analysis of volatile compounds. Mandarin hybrids without *Poncirus* introgression (green), orange varieties (orange), pummelo (pink), tangelo (pink), grapefruit (blue), *P. trifoliata* and *Poncirus* hybrids (purple) are on the y-axis and relative volatiles are on the x-axis. Darker red color indicates higher abundance of each compound and darker blue color indicates lower abundance. Harvest dates follow genotype name. (For interpretation of the references to color in this figure legend, the reader is referred to the web version of this article.)

Volatile profiles of the juices from the genotypes in this study were analyzed via headspace-SPME-GC/MS. Relative abundances, presented as a 2-way hierarchical clustering analysis, for each genotype are shown in Fig. 1. According to their volatile profiles, *P. trifoliata* samples (large and small flower) were in a distinct group from the other citrus accessions. Consistent with a previous report, *P. trifoliata* had large abundances of many volatiles identified in this study, especially sesquiterpenes, esters, and some monoterpenes (Deterre et al., 2021). *Poncirus* hybrids, ‘US119’ and FF-1-85-124, had volatile profiles more similar to the *Citrus* or mandarin hybrid genotypes without *Poncirus* introgression than the pure *P. trifoliata* juice. FF-1-85-124 and FF-5-6-36 clustered together due to their very high abundance of monoterpenes (Fig. 1). In addition, FF-5-6-36 had high abundance of the high molecular weight aldehydes octanal, nonanal and decanal. The remaining *Citrus* or mandarin hybrid genotypes clustered together and had some characteristic hot spots on the heat map. For example, the orange varieties had a higher abundance of esters while FF-1-84-2, ‘Florida Red’, ‘Melogold’, and FTP-6-32-67 had a high abundance of the aldehydes pentanal, hexanal and heptanal. ‘Cape Croen Skill’, which was too bitter to serve to panelists had an overall lack of volatile compounds, with the exception of (*E*)-2-pentenal and 1-pentanol (Fig. 1). (*E*)-2-Pentenal has a pungent, apple-like odor (Rychlik et al., 1998) and 1-pentanol (amyl alcohol) was reported to have a bitter taste (van Gemert, 2003).

### 3.3 Correlating bitterness with volatile compounds

**Fig. 2:**
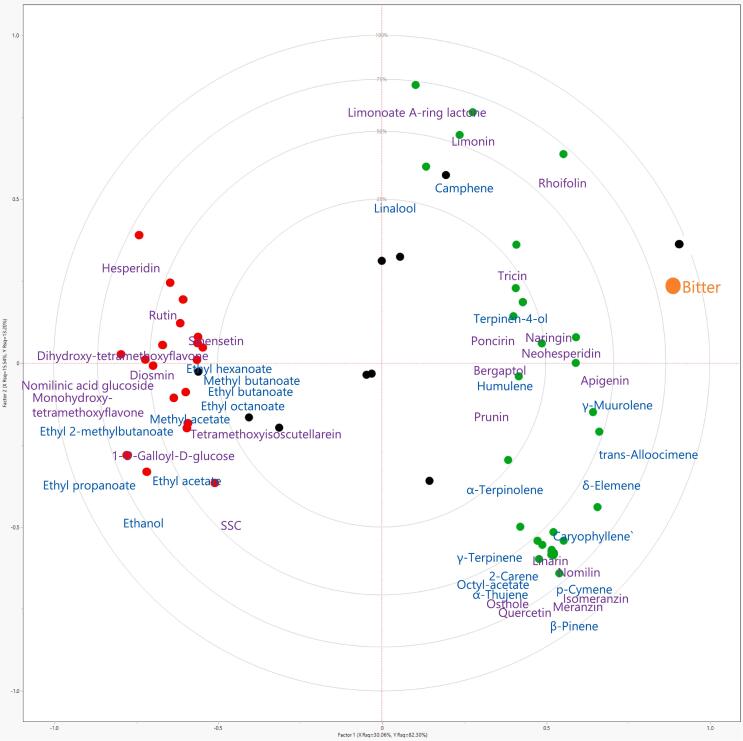
Correlation plot for partial least squares of bitter. Compounds with a green dot have a positive correlation with bitterness while compounds with a red dot have a negative correlation. Volatile compounds are colored blue while non-volatile compounds are colored purple. Black dots correspond to individual genotypes. (For interpretation of the references to color in this figure legend, the reader is referred to the web version of this article.)

**Fig. 3:**
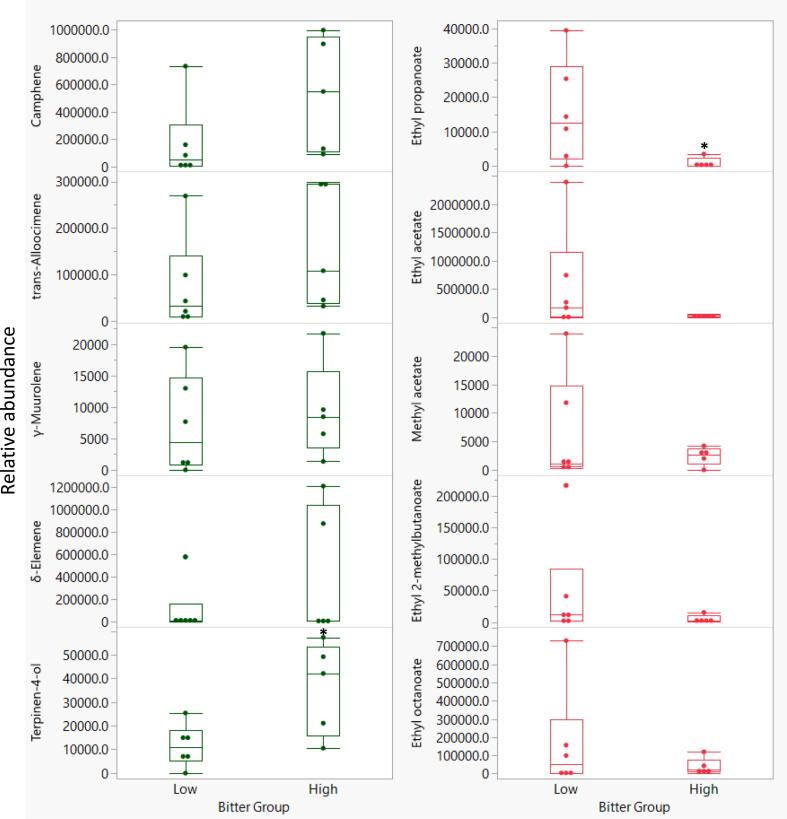
Relative abundances of key volatile compounds in low and high bitterness groups. The low bitterness group included ‘US 119’, ‘Wekiwa’, FF-1-84-2, FTP-6-32-67, and ‘Valencia’ while the high bitterness group included ‘Glen Navel’, FF-1-5-35 (harvested in 12/2021 and 1/2022), FF-1-85-124, and ‘Melogold’. Compounds that are more abundant in the high bitter group are indicated in green box plots while compounds that are less abundant in the high bitter group are indicated in red box plots. Asterisks indicate *P*-values <0.05 based on Student's *t-*tests of the difference in means between the low and high bitter groups. (For interpretation of the references to color in this figure legend, the reader is referred to the web version of this article.)

While there are very few reports of volatiles directly influencing bitterness perception, such as *cis*-3-hexen-1-ol in olive oil (Caporale et al., 2004), we investigated how volatile abundances correlated with the bitterness ratings in citrus genotypes herein. In Fig. S1, the PCA biplot shows the relationships between volatile compounds and bitterness ratings. Principal components (PC) 1 and 2 accounted for 25.6 % and 19.9 % of the variation, respectively. The compounds that were the most positively correlated (*r* > 0.3, Table S2, Fig. S1) or negatively correlated (*r* < −0.45, Table S2, Fig. S1) to bitterness were subjected to partial least squares analysis and the correlation plot is shown in Fig. 2. The closer the x-variable point is to 1 in the correlation plot, the more correlated the compound is to bitterness (y-variable). The volatile compounds that were the most positively correlated with bitterness were camphene (*r* = 0.43, Table S2), γ-muurolene (0.46), *trans*-alloocimene (0.49), terpinene-4-ol (0.52), and δ-elemene (0.61). These results are consistent with a previous study, where bitterness in *Citrus* hybrids with/without *Poncirus* introgression, was correlated to monoterpenes, camphene, α-thujene, and terpinene-4-ol (Jeffries et al., 2024). This is interesting as the genotypes herein are more diverse and include pummelo, tangelo, and grapefruit. In order to focus on some of these key compounds, the genotypes were grouped into either high or low bitterness based on panelists' comments (Table 1). The low bitterness group included ‘US 119’, ‘Wekiwa’, FF-1-84-2, FTP-6-32-67, and ‘Valencia’ while the high bitterness group included ‘Glen Navel’, FF-1-5-35 (harvested in 12/2021 and 1/2022), FF-1-85-124, and ‘Melogold’. The means of the bitter intensity ratings of the low and high bitter groups were significantly different (Student's *t-*test, *P* = 0.0002), indicating appropriate grouping. As shown in Fig. 3, the compounds positively correlated with bitterness (green box plots) had trends of being more abundant in the high bitterness group compared to the low bitterness group while those negatively correlated with bitterness (red box plots) had trends of being less abundant in the high bitterness group. However, the difference in means was only significant for two of these compounds, terpinen-4-ol and ethyl propanoate (Student's *t-*test, *P* < 0.05; Table S3).

As some compounds can act as a bitterness blocker or decrease bitterness by increasing perceived sweetness, the volatile compounds that were the most negatively correlated with bitterness were also included in Fig. 2. The volatile compounds that were the most negatively correlated with bitterness were ethyl propanoate (*r* = −0.78, Table S2), ethanol (−0.72), ethyl acetate (−0.60), methyl acetate (−0.58), ethyl 2-methylbutanoate (−0.56), ethyl octanoate (−0.46), ethyl hexanoate (−0.44), methyl butanoate (−0.44), and ethyl butanoate (−0.44). Recently, it was reported that some of these esters are important for perceiving orange flavor over mandarin flavor (Fan et al., 2024). It is possible that panelists perceived samples with more orange flavor as being less bitter since some citrus aromas are fruity and are often associated with sweetness. Preliminary studies selected five esters as sweetness enhancers and tested in model solutions (Bartoshuk et al., 2018; unpublished data).

### 3.4 Non-volatile profiling of genotypes

**Fig. 4:**
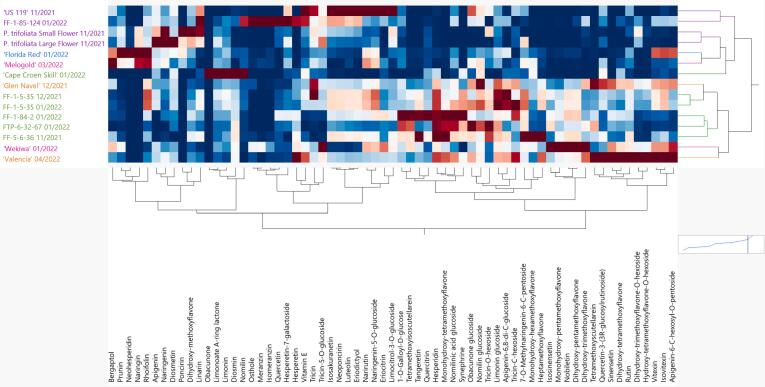
Hierarchical clustering (2-way) analysis of non-volatile compounds. Mandarin hybrids without *Poncirus* introgression (green), orange varieties (orange), pummelo (pink), tangelo (pink), grapefruit (blue), *P. trifoliata* and *Poncirus* hybrids (purple) are on the y-axis and relative non-volatiles are on the x-axis. Darker red color indicates higher abundance of each compound and darker blue color indicates lower abundance. Harvest dates follow genotype name. (For interpretation of the references to color in this figure legend, the reader is referred to the web version of this article.)

Non-volatile chemical profiles of the genotypes in this study included soluble solids content (SSC), titratable acidity (% TA), flavonoids, limonoids, coumarins, and a fat-soluble vitamin. It is well-known that SSC values are associated with juice sweetness, % TA values are associated with sourness, and limonoids (limonin and nomilin) are associated with bitterness (Deterre et al., 2023). From the genotypes in this study, the *P. trifoliata* juices (small and large flower) had the lowest SSC/TA values while ‘Cape Croen Skill’ had the largest SSC/TA value due to its extremely low titratable acidity (Table 2). The selections that were tasted for bitterness had SSC/TA ranging from 9.75 (the lowest for FF-1-85-124) to 29.46 (FTP-6-32-67) and were within typical citrus values for SSC and TA. To identify compounds other than limonin or nomilin contributing to bitterness, a widely targeted LC-MS/MS method was developed to identify and quantify 65 non-volatile compounds. Compounds were identified with two LC-MS/MS transitions based on authentic standards and literature resources. Characteristic precursor and product ions at specified collision energies were optimized for each compound (Table S1). Hierarchical clustering analysis of the genotypes based on the 65 compounds is shown in Fig. 4. The genotypes had vastly different profiles, with *Poncirus* hybrids clustering closest together (Fig. 4). Another cluster was apparent including *P. trifoliata* (large and small flower), ‘Florida Red’ (grapefruit), and ‘Melogold’ (pummelo hybrid). The mandarin hybrids without *Poncirus* introgression clustered together with the oranges and ‘Wekiwa’ (tangelo). ‘Cape Croen Skill’ was not paired with any other genotype.

*P. trifoliata* had high relative amounts of apigenin, naringenin, diosmetin, poncirin, tricin-5-O-glucoside, dihydroxy-methoxyflavone, naringin, and linarin. Consistently, *P. trifoliata* has been shown to have high concentrations of poncirin, naringin, linarin, and rhoifolin (Mou et al., 2021). *Poncirus* hybrids did not have large abundances of these compounds, except linarin (Fig. 4). Like their volatile profiles, *P. trifoliata* and *Poncirus* hybrids do not have similar flavonoid/limonoids profiles. *Poncirus* hybrids had larger abundances of isosakuranetin, neoponcirin, luteolin, and eriodictyol. The *Poncirus* hybrids, *P. trifoliata*, grapefruit, and the pummelo hybrid had lower abundances of polymethoxylated flavones (PMFs) than the oranges and the mandarin hybrids without *Poncirus* introgression. The differences between *P. trifoliata* and *Poncirus* hybrids are indicative of high genetic heterozygosity and complex admixture of mandarin, pummelo, and *P. trifoliata* alleles resulting in distinct groups that are different than pure *P. trifoliata*. The orange cultivars, ‘Glen Navel’ and ‘Valencia’, were surprisingly not clustered together, even though they were both highly abundant in PMFs, apigenin glycosides, and quercetin-3-(3R-glucosylrutinoside). However, ‘Glen Navel’ had high amount of tricin and nomilin glucoside in comparison with ‘Valencia’, and ‘Valencia’ had high amount of hesperetin in comparison with ‘Glen Navel’. Importantly, quercetin-3-(3R-glucosylrutinoside) was recently shown to be correlated with orange flavor (Jeffries et al., 2024). ‘Wekiwa’, a tangelo, interestingly clustered closest with ‘Valencia’ according to their flavonoid/limonoids profiles (Fig. 4). Unlike ‘Florida Red’ or ‘Melogold’, ‘Wekiwa’ had very high abundances of the PMFs, isosinensetin, monohydroxy-pentamethoxyflavone, nobiletin, dihydroxy-pentamethoxyflavone, and dihydroxy-trimethoxyflavone. Interestingly, ‘Valencia’ and ‘Wekiwa’ had high abundances of hesperetin (3′,5,7-Trihydroxy-4′-methoxyflavanone), a compound shown to be a natural sweetener in citrus (Z. Wang et al., 2022).

In a recent study of delayed bitterness, a rarely identified citrus compound, tricin-C-hexoside, increased dramatically with storage of juice at −20 °C (Jeffries et al., 2024). Delayed bitterness was not studied herein; however, differences in bitterness with maturity can be seen as FF-1-5-35 was harvested twice with one month between harvests. From December to January, the bitterness intensity dropped from 10.2 to 8.4 (Table 1). This decrease in bitterness was accompanied with a decrease in limonin and an increase in its tasteless glycoside, limonin glucoside (Fig. 4). This reaction, proposed to be catalyzed by limonoid UDP-glucosyltransferase (Kita et al., 2000), is a well-known natural process as fruit mature (Herman et al., 1991). More recently, the direct substrates of this enzyme were questioned (Cui et al., 2020) and future work is needed to determine limonoid biosynthetic pathways. Between December and January, there was also a large decrease in the polymethoxylated flavone, tricin, and a large increase in its glycoside, tricin-C-hexoside. While there have been no reports on the tastes of tricin or tricin-C-hexoside, it should be the focus of future studies in citrus bitterness.

### 3.5 Correlating bitterness with non-volatile compounds

**Fig. 5:**
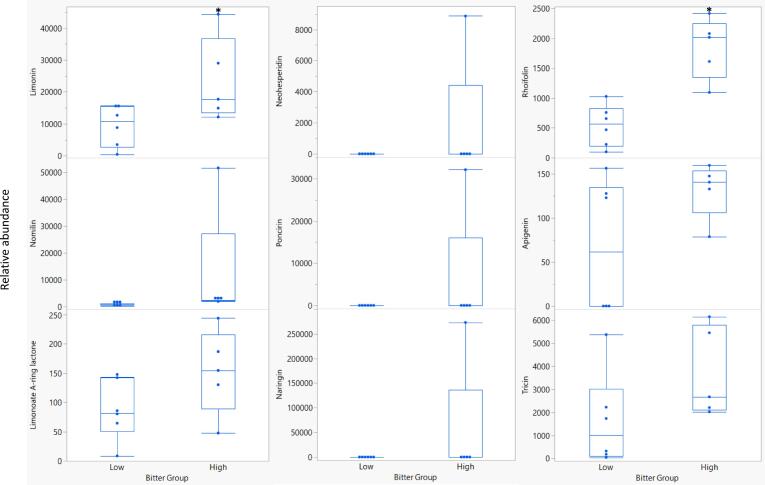
Relative abundances of key non-volatile compounds in low and high bitterness groups. The low bitterness group included ‘US 119’, ‘Wekiwa’, FF-1-84-2, FTP-6-32-67, and ‘Valencia’ while the high bitterness group included ‘Glen Navel’, FF-1-5-35 (harvested in 12/2021 and 1/2022), FF-1-85-124, and ‘Melogold’. Asterisks indicate *P*-values <0.05 based on Student's *t-*tests of the difference in means between the low and high bitter groups.

In addition to the volatile compounds, the non-volatile compounds that were the most positively correlated (*r* > 0.3, Table S4, Fig. S2) or negatively correlated (*r* < −0.45, Table S4, Fig. S2) to bitterness were subjected to partial least squares analysis and the correlation plot is shown in Fig. 2. In Fig. S2, the PCA biplot shows the relationships between volatile compounds and bitterness ratings. Principal components (PC) 1 and 2 accounted for 25.2 % and 18.8 % of the variation, respectively. The non-volatile compounds that were the most correlated with bitterness were rhoifolin (*r* = 0.77, Table S4), limonin (0.57), apigenin (0.52), neohesperidin (0.46), poncirin (0.46), naringin (0.46), limonoate A-ring lactone (0.42), tricin (0.41), and nomilin (0.41). Grouping the genotypes into either low or high bitterness, Fig. 5 shows that compounds positively correlated with bitterness had trends of being more abundant in the high bitterness group compared to the low bitterness group. Of these compounds, rhoifolin, apigenin, and tricin have not been identified as bitter compounds in citrus while neohesperidin, poncirin, and naringin were only present in the pummelo, ‘Melogold’. Importantly, rhoifolin had a stronger correlation (r = 0.77, Table S4) with bitterness than the very well-known citrus bitter compound, limonin (*r* = 0.57). As indicated in Fig. 5, the difference in means between the low and high bitterness groups were significant only for rhoifolin (*P* = 0.0013, Table S5) and limonin (*P* = 0.0463, Table S5). The individual taste of rhoifolin (synonym apigenin-7-O-neohesperidoside) has not been reported, but should be analyzed with a sensory panel in future work. Recently, it was reported that rhoifolin, naringin, poncirin, diosmin, neoponcirin, and narirutin were responsible for bitterness in a traditional Chinese medicine formula, Runchang-Tongbian (N. Li et al., 2024). While it is known that the presence of the neohesperidose moity in a flavonoid glycoside is not sufficient to produce bitterness on its own (Horowitz et al., 1963), there are a number of flavonoid-neohesperidosides that are bitter, such as naringin. The aglycone of rhoifolin, apigenin, had a stronger positive correlation with bitterness than the other known bitter compounds in citrus, neohesperidin, poncirin, and naringin (Table S4). Using modeling, apigenin was shown to activate hTAS2R14 and hTAS2R39 bitter receptors (Roland et al., 2013). Additionally, it was shown using bitter receptor modeling that the substitution pattern of the isoflavonoid was of higher importance for receptor activation than its backbone structure, such as flavone versus flavanone. In a recent study, tricin and apigenin (in addition to quercetin 3-(3R-glucosylrutinoside and tricin-5-*O*-glucoside) were negatively correlated with mandarin flavor (Jeffries et al., 2024).

The nonvolatile compounds that were the most negatively correlated with bitterness were monohydroxy-tetramethoxyflavone (*r* = −0.65, Table S4 and Fig. S2), 1-*O*-galloyl-d-glucose (*r* = −0.58), tetramethoxyisoscutellarein (*r* = −0.57), nomilinic acid glucoside (*r* = −0.56), and SSC (r = −0.56). Monohydroxy-tetramethoxyflavone, 1-*O*-galloyl-d-glucose, and tetramethoxyisoscutellarein are not known to be sweetness enhancers or bitterness blockers. Nomilinic acid glucoside is the tasteless form of the well-known bitter limonoid, nomilin, explaining its negative correlation with bitterness. SSC being negatively correlated with bitterness is due to the sugar's sweetness suppressing bitterness.

## 4 Conclusion

In conclusion, a diverse selection of citrus juices was comprehensively analyzed via sensory and chemical methods. The genotypes in this study were rated across a spectrum of low to high bitterness intensities. Correlations between the bitterness intensities and the chemical components gave insights into the complex nature of citrus juices. Given reports that volatile compounds can affect taste, both volatile and non-volatile components were analyzed. Certain monoterpenes, such as camphene and *trans*-alloocimene, along with some sesquiterpenes, including γ-muurolene and δ-elemene, were positively correlated with bitterness. Esters, which contribute to the orange flavor, were strongly negatively correlated with bitterness.

Citrus limonoids, specially limonin and nomilin, are well-known contributors to bitterness. A widely targeted LC-MS/MS method was developed to identify and quantify 65 non-volatile compounds to determine whether compounds other than limonin or nomilin contribute to bitterness in citrus. Rhoifolin, a neohesperidose-conjugated flavone, was found to be more strongly correlated with bitterness than limonin. In addition to the known contributors to bitterness (limonin, nomilin, neohesperidin, and poncirin), the aglycone of rhoifolin, apigenin, and another aglycone, tricin, were also correlated with bitterness. Their bitterness activity should be confirmed with tasting of pure compounds. It is well-known that the flavor of citrus juice is incredibly complex, and off-flavors and undesirable bitterness are not caused by a single compound. Nevertheless, compounds found herein can be considered as negative biomarkers in citrus breeding. Thus, future breeding efforts may need to target multiple biosynthetic pathways to reduce bitterness, especially in the era of citrus greening.

## CRediT authorship contribution statement

**Kristen A. Jeffries:** Writing – review & editing, Writing – original draft, Visualization, Methodology, Investigation, Formal analysis, Data curation, Conceptualization. **Zhen Fan:** Writing – review & editing, Formal analysis, Data curation. **Matthew Mattia:** Writing – review & editing, Resources, Methodology, Investigation, Conceptualization. **Ed Stover:** Writing – review & editing, Resources. **Elizabeth Baldwin:** Writing – review & editing. **John A. Manthey:** Writing – review & editing, Resources, Conceptualization. **Andrew Breksa:** Writing – review & editing, Resources, Conceptualization. **Jinhe Bai:** Writing – review & editing, Resources, Investigation, Conceptualization. **Anne Plotto:** Writing – review & editing, Supervision, Resources, Project administration, Methodology, Investigation, Data curation, Conceptualization.

## Declaration of competing interest

The authors declare that they have no known competing financial interests or personal relationships that could have appeared to influence the work reported in this paper.

## Data Availability

Data will be made available on request.

## Appendix A Supplementary data

Supplementary material 1: Supplementary Table 1: LC-MS/MS dMRM transitions and compound specific parameters Image 1

Supplementary material 2: Supplementary Table 2. Correlations between bitterness intensity and volatile components. Blue coloring corresponds to a more positive correlation while red coloring corresponds to a more negative correlation. Image 2

Supplementary material 3: Supplementary Table 3. Mean relative abundances of volatile compounds for low and high bitterness groups. Image 3

Supplementary material 4: Supplementary Table 4. Correlations between bitterness intensity and nonvolatile components. Blue coloring corresponds to a more positive correlation while red coloring corresponds to a more negative correlation. Image 4

Supplementary material 5: Table S5. Mean relative abundances of non-volatile compounds for low and high bitterness groups. Image 5

Supplementary material 6: **Figure S1: Principal components analysis (PCA) of bitterness intensity and volatile compounds.** Mandarin hybrids without *Poncirus* introgression are green, orange varieties are orange, pummelo is pink, tangelo is pink, *P. trifoliata* and *Poncirus* hybrids are purple. Harvest dates follow genotype name. **Figure S2: Principal components analysis (PCA) of bitterness intensity and nonvolatile compounds.** Mandarin hybrids without *Poncirus* introgression are green, orange varieties are orange, pummelo is pink, tangelo is pink, *P. trifoliata* and *Poncirus* hybrids are purple. Harvest dates follow genotype name. Image 6
