# Supplementary material for: Flavonoid contributors to bitterness in juice from Citrus and Citrus hybrids with/without Poncirus trifoliata in their pedigree
Source: Food Chem X. 2025 Feb 18;26:102289. doi: 10.1016/j.fochx.2025.102289 (PMC11905843; doi:10.1016/j.fochx.2025.102289)
Supplement: Supplementary file 1 — Supplementary material 1: Supplementary Table 1: LC-MS/MS dMRM transitions and compound specific parameters [file mmc1.docx]

**Supplementary Table 1:** **LC-MS/MS dMRM transitions and compound specific parameters**

| **Compound Name** | **Precursor Ion^a^** | **Product Ion** | **Fragmentor (V)** | **Collision Energy (V)** | **Retention Time (min)** | **Polarity** | **Identification Level^b^** |
| --- | --- | --- | --- | --- | --- | --- | --- |
| Synephrine | **168.1** | **135** | **170** | **20** | **0.814** | **Positive** | A |
| Synephrine | 168.1 | 107 | 170 | 20 | 0.814 | Positive |  |
| Bergaptol | **203** | **147** | **120** | **18** | **5.463** | Positive | A |
| Osthole | **245.2** | **189** | **90** | **15** | **8.777** | **Positive** | A |
| Osthole | 245.2 | 159.2 | 90 | 15 | 8.777 | Positive |  |
| Meranzin | **261** | **189** | **120** | **16** | **6.772** | **Positive** | B |
| Isomeranzin | **261.1** | **189** | **118** | **10** | **6.970** | **Positive** | A |
| Isomeranzin | 261.1 | 131 | 118 | 30 | 6.970 | Positive |  |
| Apigenin | **271.1** | **153** | **170** | **34** | **5.964** | **Positive** | A |
| Apigenin | 271.1 | 119.1 | 170 | 34 | 5.964 | Positive |  |
| Naringenin | 273.1 | 153 | 145 | 22 | 5.898 | Positive | A |
| Naringenin | **273.1** | **147.1** | **145** | **18** | **5.898** | **Positive** |  |
| Isosakuranetin | 285.1 | 243.1 | 190 | 22 | 5.388 | Negative | A |
| Isosakuranetin | **285.1** | **164** | **190** | **26** | **5.388** | **Negative** |  |
| Dihydroxy-methoxyflavone | **285.1** | **270** | **170** | **20** | **5.365** | **Positive** | B |
| Dihydroxy-methoxyflavone | 285.1 | 131.1 | 170 | 20 | 5.365 | Positive |  |
| Luteolin | **287.1** | **153** | **174** | **34** | **5.391** | **Positive** | A |
| Luteolin | 287.1 | 135 | 174 | 34 | 5.391 | Positive |  |
| Diosmetin | 301.1 | 286.1 | 165 | 26 | 6.134 | Positive | A |
| Diosmetin | **301.1** | **258.1** | **165** | **38** | **6.134** | **Positive** |  |
| Hesperetin | 303.1 | 177 | 110 | 18 | 6.193 | Positive | A |
| Hesperetin | **303.1** | **153** | **110** | **26** | **6.193** | **Positive** |  |
| Quercetin | **303.1** | **153** | **170** | **38** | **5.430** | **Positive** | A |
| Quercetin | 303.1 | 69.1 | 170 | 50 | 5.430 | Positive |  |
| 1-O-Galloyl-D-Glucose | **331** | **169** | **170** | **20** | **3.650** | **Negative** | B |
| Tricin | 331.1 | 316.1 | 170 | 30 | 5.968 | Positive | A |
| Tricin | **331.1** | **301** | **170** | **30** | **5.968** | **Positive** |  |
| Tetramethoxyisoscutellarein | **343** | **313** | **90** | **18** | **6.977** | **Positive** | B |
| Tetramethoxyscutellarein | 343.1 | 299.1 | 133 | 26 | 7.473 | Positive | A |
| Tetramethoxyscutellarein | **343.1** | **282.1** | **133** | **26** | **7.473** | **Positive** |  |
| Dihydroxy-trimethoxyflavone 1 | 345.1 | 330.1 | 170 | 20 | 7.472 | Positive | B |
| Dihydroxy-trimethoxyflavone 1 | **345.1** | **315.1** | **170** | **20** | **7.472** | **Positive** |  |
| Monohydroxy-tetramethoxyflavone | **359.1** | **344.1** | **170** | **20** | **7.956** | **Positive** | B |
| Monohydroxy-tetramethoxyflavone | 359.1 | 329.1 | 170 | 20 | 7.956 | Positive |  |
| Sinensetin | 373.1 | 343.1 | 150 | 30 | 6.966 | Positive | A |
| Sinensetin | **373.1** | **312.1** | **150** | **26** | **6.966** | **Positive** |  |
| Tangeretin | 373.1 | 358.1 | 150 | 18 | 7.959 | Positive | A |
| Tangeretin | **373.2** | **343** | **120** | **26** | **7.959** | **Positive** |  |
| Isosinensetin | **373.2** | **343** | **92** | **26** | **6.518** | **Positive** | A |
| Isosinensetin | 373.2 | 312.1 | 92 | 26 | 6.518 | Positive |  |
| Dihydroxy-tetramethoxyflavone | **375.1** | **345.1** | **170** | **15** | **6.965** | **Positive** | B |
| Dihydroxy-tetramethoxyflavone | 375.1 | 312.3 | 170 | 15 | 6.965 | Positive |  |
| Monohydroxy-pentamethoxyflavone | 389.1 | 359.1 | 170 | 30 | 8.381 | Positive | B |
| Monohydroxy-pentamethoxyflavone | **389.1** | **341.1** | **170** | **30** | **8.381** | **Positive** |  |
| Nobiletin | 403.1 | 388.1 | 158 | 22 | 7.452 | Positive | A |
| Nobiletin | **403.1** | **373.1** | **158** | **30** | **7.452** | **Positive** |  |
| Dihydroxy-pentamethoxyflavone | **405.1** | **375.1** | **170** | **20** | **7.449** | **Positive** | B |
| Dihydroxy-pentamethoxyflavone | 405.1 | 347.1 | 170 | 20 | 7.449 | Positive |  |
| 7-O-Methylnaringenin-6-C-pentoside | 419.1 | 383.1 | 170 | 30 | 3.622 | Positive | B |
| 7-O-Methylnaringenin-6-C-pentoside | **419.1** | **365.1** | **170** | **30** | **3.622** | **Positive** |  |
| Monohydroxy-hexamethoxyflavone | 419.1 | 404.1 | 170 | 30 | 7.974 | Positive | B |
| Monohydroxy-hexamethoxyflavone | **419.1** | **389.1** | **170** | **30** | **7.974** | **Positive** |  |
| Mangiferin | 423.1 | 303 | 122 | 18 | 3.412 | Positive | A |
| Mangiferin | **423.1** | **273** | **122** | **22** | **3.412** | **Positive** |  |
| Vitamin E | **431.4** | **165** | **130** | **18** | **18.580** | **Positive** | A |
| Prunin | **433.1** | **271.1** | **130** | **10** | **4.579** | **Negative** | A |
| Prunin | 433.1 | 151 | 130 | 34 | 4.579 | Negative |  |
| Vitexin | 433.1 | 313.1 | 170 | 20 | 4.096 | Positive | A |
| Vitexin | **433.1** | **283.1** | **170** | **20** | **4.096** | **Positive** |  |
| Heptamethoxyflavone | **433.3** | **403.1** | **170** | **20** | **7.775** | **Positive** | A |
| Heptamethoxyflavone | 433.3 | 385.1 | 170 | 20 | 7.775 | Positive |  |
| Isovitexin | 433.4 | 397.1 | 170 | 20 | 3.976 | Positive | A |
| Isovitexin | **433.4** | **313.1** | **170** | **20** | **3.976** | **Positive** |  |
| Naringenin 5-O-glucoside | **435.1** | **273.1** | **170** | **30** | **4.401** | **Positive** | A |
| Naringenin 5-O-glucoside | 435.1 | 177.1 | 170 | 30 | 4.401 | Positive |  |
| Quercitrin | **447.1** | **301.1** | **170** | **22** | **4.489** | **Negative** | A |
| Quercitrin | 447.1 | 300 | 170 | 26 | 4.489 | Negative |  |
| Obacunone | **455.2** | **409.2** | **160** | **25** | **8.017** | **Positive** | A |
| Hesperetin-7-galactoside | **463.1** | **301.1** | **114** | **14** | **4.712** | **Negative** | A |
| Hesperetin-7-galactoside | 463.1 | 286.1 | 114 | 34 | 4.712 | Negative |  |
| Limonin | 471.2 | 425.2 | 135 | 19 | 7.065 | Positive | A |
| Limonin | **471.2** | **161.2** | **135** | **30** | **7.065** | **Positive** |  |
| Limonoate A-ring lactone | **487.2** | **457.2** | **154** | **18** | **6.407** | **Negative** | A |
| Limonoate A-ring lactone | 487.2 | 369.2 | 154 | 26 | 6.407 | Negative |  |
| Limonoate A-ring lactone | 487.2 | 337.2 | 154 | 22 | 6.407 | Negative |  |
| Tricin C-hexoside | **493.1** | **475.1** | **170** | **20** | **4.993** | **Positive** | B |
| Tricin C-hexoside | 493.1 | 457.1 | 170 | 20 | 4.993 | Positive |  |
| Tricin O-hexoside | **493.1** | **331.1** | **170** | **20** | **5.340** | **Positive** | B |
| Tricin O-hexoside | 493.1 | 316.1 | 170 | 30 | 5.340 | Positive |  |
| Tricin-5-O-glucoside | 493.1 | 270 | 96 | 40 | 4.380 | Positive | A |
| Tricin-5-O-glucoside | **493.1** | **331.1** | **96** | **10** | **4.380** | **Positive** |  |
| Tricin-5-O-glucoside | 493.1 | 315.1 | 96 | 40 | 4.380 | Positive |  |
| Dihydroxy-trimethoxyflavone-O-hexoside | **507.1** | **345.1** | **170** | **30** | **4.359** | **Positive** | B |
| Dihydroxy-trimethoxyflavone-O-hexoside | 507.1 | 330.1 | 170 | 30 | 4.359 | Positive |  |
| Nomilin | 515.3 | 411.2 | 135 | 14 | 7.500 | Positive | A |
| Nomilin | **515.3** | **161.1** | **135** | **25** | **7.500** | **Positive** |  |
| Hydroxy-tetramethoxyflavone-O-hexoside | **521.1** | **359.1** | **170** | **30** | **4.853** | **Positive** | B |
| Hydroxy-tetramethoxyflavone-O-hexoside | 521.1 | 344.1 | 170 | 30 | 4.853 | Positive |  |
| Limocitrol 3-O-glucoside | **539.1** | **377.1** | **170** | **30** | **5.614** | **Positive** | B |
| Limocitrol 3-O-glucoside | 539.1 | 128.1 | 170 | 30 | 5.614 | Positive |  |
| Apigenin 6-C-hexosyl-O-pentoside | 565.1 | 433.1 | 170 | 30 | 4.032 | Positive | B |
| Apigenin 6-C-hexosyl-O-pentoside | **565.1** | **313.1** | **170** | **30** | **4.032** | **Positive** |  |
| Naringin | **579.2** | **271.1** | **120** | **38** | **4.498** | **Negative** | A |
| Naringin | 579.2 | 151 | 120 | 40 | 4.498 | Negative |  |
| Narirutin | **579.2** | **271.1** | **170** | **22** | **4.389** | **Negative** | A |
| Narirutin | 579.2 | 151 | 170 | 42 | 4.389 | Negative |  |
| Rhoifolin | 579.2 | 271.1 | 170 | 30 | 4.156 | Positive | A |
| Rhoifolin | **579.2** | **85** | **170** | **30** | **4.156** | **Positive** |  |
| Neoponcirin | 593.2 | 309.1 | 160 | 26 | 5.386 | Negative | A |
| Neoponcirin | **593.2** | **285.1** | **160** | **26** | **5.386** | **Negative** |  |
| Poncirin | 593.2 | 327.1 | 170 | 30 | 5.495 | Negative | A |
| Poncirin | **593.2** | **285.1** | **170** | **34** | **5.495** | **Negative** |  |
| Linarin | **593.3** | **285.1** | **170** | **20** | **5.290** | **Positive** | A |
| Linarin | 593.3 | 127 | 170 | 20 | 5.290 | Positive |  |
| Eriocitrin | 595.2 | 287 | 120 | 36 | 4.043 | Negative | A |
| Eriocitrin | **595.2** | **151** | **170** | **38** | **4.043** | **Negative** |  |
| Apigenin-6,8-di-C-glucoside | **595.2** | **457** | **170** | **20** | **3.487** | **Positive** | B |
| Apigenin-6,8-di-C-glucoside | 595.2 | 325 | 170 | 20 | 3.487 | Positive |  |
| Diosmin | **607.2** | **299.1** | **170** | **22** | **4.556** | **Negative** | A |
| Diosmin | 607.2 | 284.1 | 170 | 50 | 4.556 | Negative |  |
| Hesperidin | **609.2** | **301.1** | **110** | **26** | **4.623** | **Negative** | A |
| Neohesperidin | **609.2** | **301.1** | **170** | **34** | **4.714** | **Negative** | A |
| Neohesperidin | 609.2 | 286.1 | 170 | 46 | 4.714 | Negative |  |
| Rutin | **609.2** | **300** | **120** | **23** | **4.022** | **Negative** | A |
| Obacunone Glucoside | **633.2** | **427.2** | **205** | **30** | **5.177** | **Negative** | A |
| Obacunone Glucoside | 633.2 | 101 | 205 | 38 | 5.177 | Negative |  |
| Limonin Glucoside | 649.2 | 605.3 | 170 | 26 | 4.249 | Negative | A |
| Limonin Glucoside | **649.2** | **443.2** | **170** | **26** | **4.249** | **Negative** |  |
| Nomilin Glucoside | 693.3 | 89.1 | 205 | 46 | 4.950 | Negative | A |
| Nomilin Glucoside | **693.3** | **59.1** | **205** | **50** | **4.950** | **Negative** |  |
| Nomilinic Acid Glucoside | 711.3 | 651.3 | 165 | 26 | 4.930 | Negative | A |
| Nomilinic Acid Glucoside | **711.3** | **607.3** | **165** | **26** | **4.930** | **Negative** |  |
| Quercetin 3-3R-glucosylrutinoside | **773.2** | **465** | **170** | **20** | **3.160** | **Positive** | B |
| Quercetin 3-3R-glucosylrutinoside | 773.2 | 303 | 170 | 20 | 3.160 | Positive |  |

^a^ Transitions used for quantification are bolded.

^b^ Identification Level A: Identified with analytical standard. Identification Level B: Identified with MRM transitions from public databases and references^1,2,3^

1. Feng, S., Niu, L., Suh, J. H., Hung, W. L., & Wang, Y. (2018, Oct 3). Comprehensive Metabolomics Analysis of Mandarins (Citrus reticulata) as a Tool for Variety, Rootstock, and Grove Discrimination. J Agric Food Chem, 66(39), 10317-10326. https://doi.org/10.1021/acs.jafc.8b03877
2. Wang, S., Yang, C., Tu, H., Zhou, J., Liu, X., Cheng, Y., Luo, J., Deng, X., Zhang, H., & Xu, J. (2017, Sep 5). Characterization and Metabolic Diversity of Flavonoids in Citrus Species. Sci Rep, 7(1), 10549. https://doi.org/10.1038/s41598-017-10970-2
3. Wang, F., Chen, L., Chen, S., Chen, H., & Liu, Y. (2021). Characterization of two closely related citrus cultivars using UPLC-ESI-MS/MS-based widely targeted metabolomics. PLoS One, 16(7), e0254759. https://doi.org/10.1371/journal.pone.0254759
