# Supplementary figures and images for: Flavonoid contributors to bitterness in juice from Citrus and Citrus hybrids with/without Poncirus trifoliata in their pedigree
Source: Food Chem X. 2025 Feb 18;26:102289. doi: 10.1016/j.fochx.2025.102289 (PMC11905843; doi:10.1016/j.fochx.2025.102289)

## Slide 1
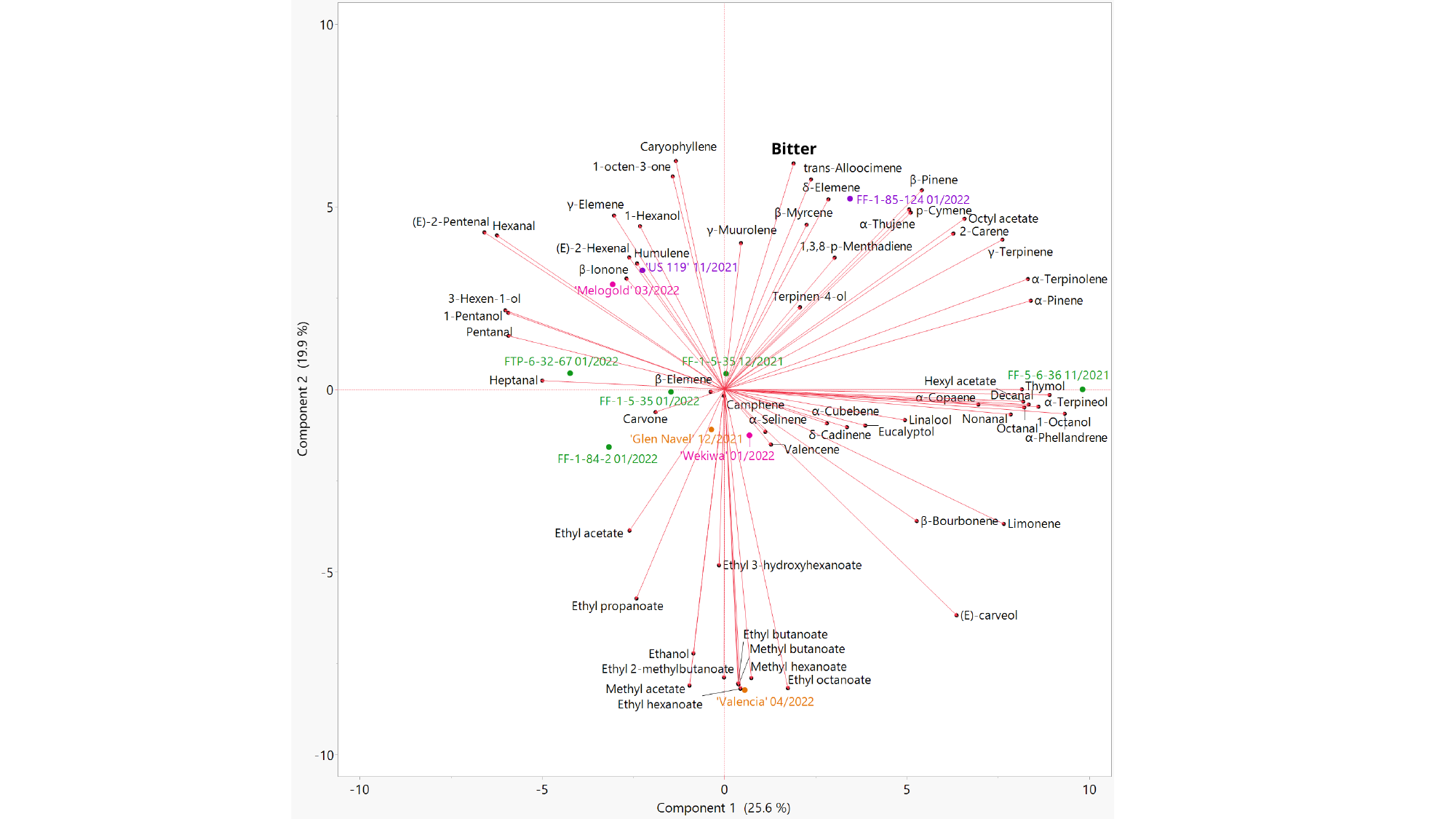

Bitter

## Slide 2
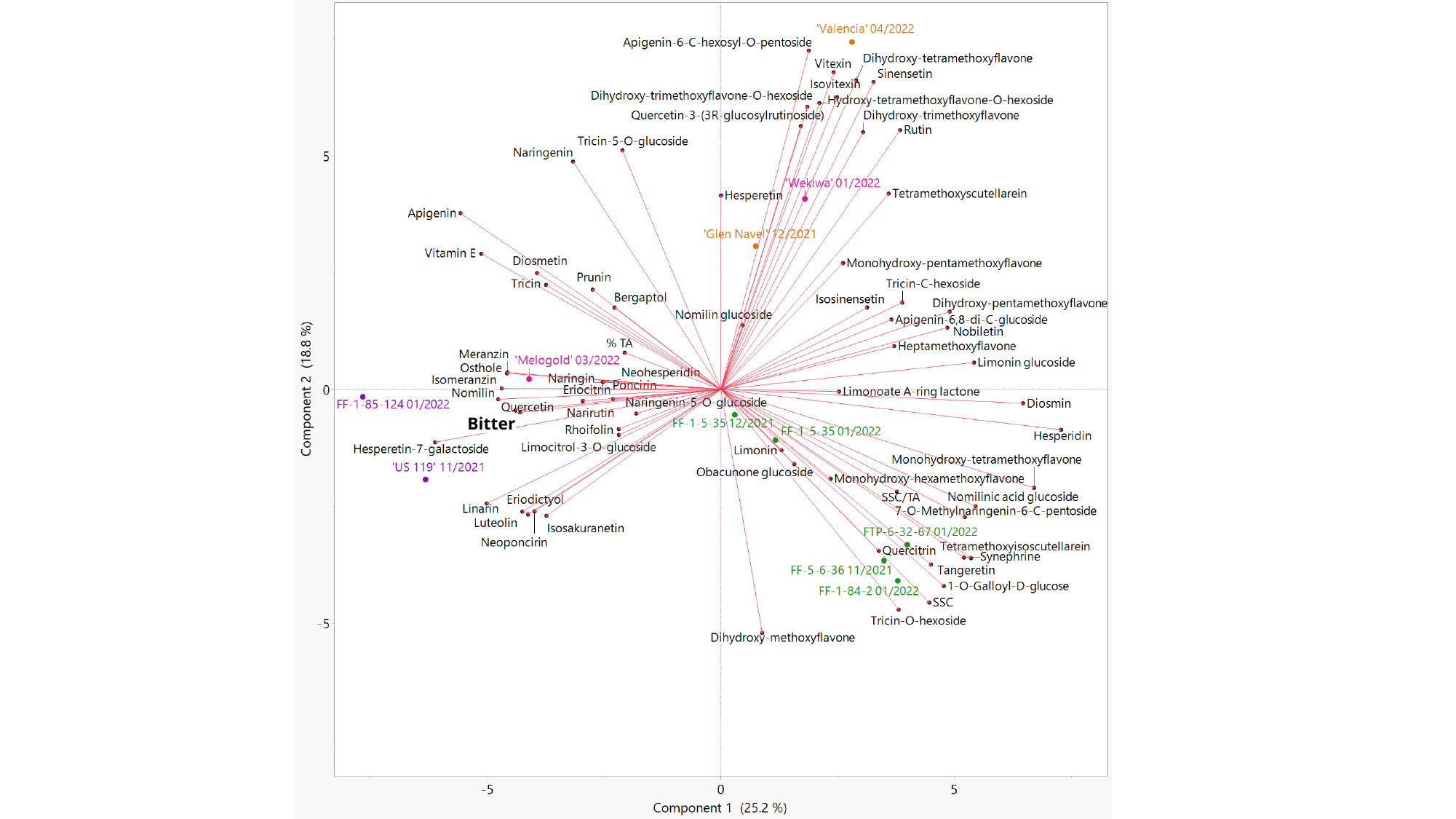

Bitter

Supplement: Supplementary file 6 — Supplementary material 6: Figure S1: Principal components analysis (PCA) of bitterness intensity and volatile compounds. Mandarin hybrids without Poncirus introgression are green, orange varieties are orange, pummelo is pink, tangelo is pink, P. trifoliata and Poncirus hybrids are purple. Harvest dates follow genotype name. Figure S2: Principal components analysis (PCA) of bitterness intensity and nonvolatile compounds. Mandarin hybrids without Poncirus introgression are green, orange varieties are orange, pummelo is pink, tangelo is pink, P. trifoliata and Poncirus hybrids are purple. Harvest dates follow genotype name. [file mmc6.pptx]
